# An empirical investigation of the benefit of increasing the temporal resolution of task-evoked fMRI data with multi-band imaging

**DOI:** 10.1007/s10334-021-00918-z

**Published:** 2021-03-25

**Authors:** Virág Darányi, Petra Hermann, István Homolya, Zoltán Vidnyánszky, Zoltan Nagy

**Affiliations:** 1 Brain Imaging Centre, Research Centre for Natural Sciences, Budapest, Hungary; 2 Laboratory for Social and Neural Systems Research, University of Zürich, Rämistrasse 100, P.O. Box 149, Zürich, Switzerland

**Keywords:** Temporal sampling rate, fMRI, Goodness-of-fit, Multi-band, SNR

## Abstract

**Objective:**

There is a tendency for reducing TR in MRI experiments with multi-band imaging. We empirically investigate its benefit for the group-level statistical outcome in task-evoked fMRI.

**Methods:**

Three visual fMRI data sets were collected from 17 healthy adult participants. Multi-band acquisition helped vary the TR (2000/1000/410 ms, respectively). Because these data sets capture different temporal aspects of the haemodynamic response (HRF), we tested several HRF models. We computed a composite descriptive statistic, H, from *β*’s of each first-level model fit and carried it to the group-level analysis. The number of activated voxels and the *t* value of the group-level analysis as well as a goodness-of-fit measure were used as surrogate markers of data quality for comparison.

**Results:**

Increasing the temporal sampling rate did not provide a universal improvement in the group-level statistical outcome. Rather, both the voxel-wise and ROI-averaged group-level results varied widely with anatomical location, choice of HRF and the setting of the TR. Correspondingly, the goodness-of-fit of HRFs became worse with increasing the sampling frequency.

**Conclusion:**

Rather than universally increasing the temporal sampling rate in cognitive fMRI experiments, these results advocate the performance of a pilot study for the specific ROIs of interest to identify the appropriate temporal sampling rate for the acquisition and the correspondingly suitable HRF for the analysis of the data.

**Supplementary Information:**

The online version contains supplementary material available at 10.1007/s10334-021-00918-z.

## Introduction

After the development of in-plane parallel imaging methods in the 1990’s, [1–3] slice-wise acceleration, a.k.a. simultaneous multi-slice (SMS) or multi-band (MB) imaging, was proposed by Larkman et al. in the early 2000’s [4] and subsequently refined by others to become a widely utilized neuroimaging method [5–7].

A reasonable supposition is that increasing the temporal sampling rate (i.e. shorter repetition time (TR)) should improve the detection of blood-oxygen-level-dependent (BOLD) signal (see for example Feinberg et al. [8]). The benefit of higher number of data points per unit time is expected from the increased degrees of freedom for statistical analysis and the ability to more appropriately sample physiological processes (e.g. cardiac pulsations) up to around 1 Hz.

Indeed, in resting-state functional magnetic resonance imaging (fMRI), MB imaging has shown utility [8, 9]. However, it is still unclear when the benefits of the higher temporal sampling outweigh the correspondingly incurred SNR loss in task-based fMRI efforts [10–12]. The SNR loss is partly due to incomplete T1 relaxation during the short TR [13]. Larger MB acceleration factors often make use of in-plane parallel imaging acceleration as well, which imparts an additional spatially variable reduction in SNR through the g-factor (although not the multi-band acceleration factor) [4, 14]. Furthermore, due to the shorter TR, temporal autocorrelations may also increase in the voxel-wise time course of fMRI data, which require careful additional processing before reliable statistical inference can be drawn [15, 16].

The physiological response to neural activity is highly variable across anatomical regions [17] as well as between participants or upon repeated scanning in a single participant [18]. The BOLD activation is usually ascertained by fitting one of several hemodynamic response functions (HRFs) to the fMRI time-series data (e.g. see chapter 14 in Friston et al. [19]). These models have a varying number of fitted variables, some of which capture different temporal aspects of the hemodynamic response. Therefore, an additional practical complexity is if and how much the additional data with the increased temporal sampling rate will impact the detection of the BOLD response while fitting these various models.

The overarching aim of this study was to empirically assess whether MB imaging data would provide a universal benefit regardless of anatomical area of the brain or the HRF used. In a realistic expectation, we hypothesized that such benefit, even if universally detectable, would likely be modulated by the anatomical location and/or the HRF model used. Therefore, we systematically tested five different HRF models in four different occipito-temporal regions of interest.

## Methods

### Participants

Each of the 21 healthy, right-handed human volunteers signed a written informed consent before undergoing a scanning session. Both the consent form and the scanning protocol were approved by the Health Registration and Training Centre (ENKK006641/2016/OTIG) and were in accordance with Helsinki Declaration of 1964 and its subsequent amendments. Four participants were excluded: one for excessive motion during the experiment, while three others had incomplete data. The remaining 17 participants (10 female/7 male) form the group described in this study. These data were utilized from a previous study with a separate aim that investigated the efficiency of MB sequences for different measurement durations [20].

### Data acquisition

#### Imaging protocol

All magnetic resonance imaging (MRI) data were collected on a 3 T Siemens Magnetom Prisma scanner (Siemens Healthcare, Erlangen, Germany) and the vendor’s 64ch receive-only head and neck coil (52 channels used for acquisition). Three different fMRI data sets were collected with 2D gradient echo echo-planar imaging (EPI) [21] with three different TRs. To increase the sampling rate of the BOLD signal (i.e. reduce TR), two of these acquisitions employed slice-wise multi-band acceleration of 4 or 6 [6]. Henceforth, the three fMRI sequences will be referred to as MB1 (i.e. without multi-band), MB4 and MB6, respectively. In an attempt to keep the signal-to-noise ratio (SNR) optimum for each TR, the flip angle (FA) was set to the Ernst angle assuming a T1 relaxation time of 1200 ms for grey matter (GM). For a fair comparison, the total acquisition time, the echo time (TE) and the voxel dimensions were kept constant across the fMRI data sets (see Table 1 for the relevant image acquisition details). The MB4 and MB6 images were reconstructed as in Cauley et al. [22] to alleviate interslice signal leakage. The order of the three MB factors was pseudo randomized and balanced across the original of 21 volunteers. The randomization remained sufficient across the 17 participants included in the study (Table 1). In addition, the MB1 sequence was used for a short functional localizer experiment that identified four regions of interest (ROIs).

**Table 1:** Sequence acquisition parameters and pseudorandomization of the order of MB factors

Parameter	MB1	MB4	MB6
TR (ms)	2000	1000	410
FA (°)	79	64	45
MB-factor	1	4	6
GRAPPA	2	N/A	N/A
Partial Fourier	N/A	7/8	7/8
Readout BW (Hz/pixel)	2170	2170	2170
Echo spacing (ms)	0.55	0.57	0.61
Image Volumes	336	672	1638
Time-series length	11m20s	11m20s	11m17s
TE (ms)	30 ms	30 ms	30 ms
Voxel size	3.0 × 3.0 × 3.0 mm^3^ (25% slice gap)	3.0 × 3.0 × 3.0 mm^3^ (25% slice gap)	3.0 × 3.0 × 3.0 mm^3^ (25% slice gap)
Num of Slices	36	36	36
Scan order 1st	6	5	6
Scan order 2nd	4	7	6
Scan order 3rd	7	5	5

The imaging protocol also included a 1.0 mm isotropic resolution 3D T1-weigthed MPRAGE sequence [23] with TR/TE/FA = 2300 ms/3.03 ms/9° and inversion time of 900 ms for anatomical normalization.

#### fMRI stimulus paradigm

**Fig. 1:**
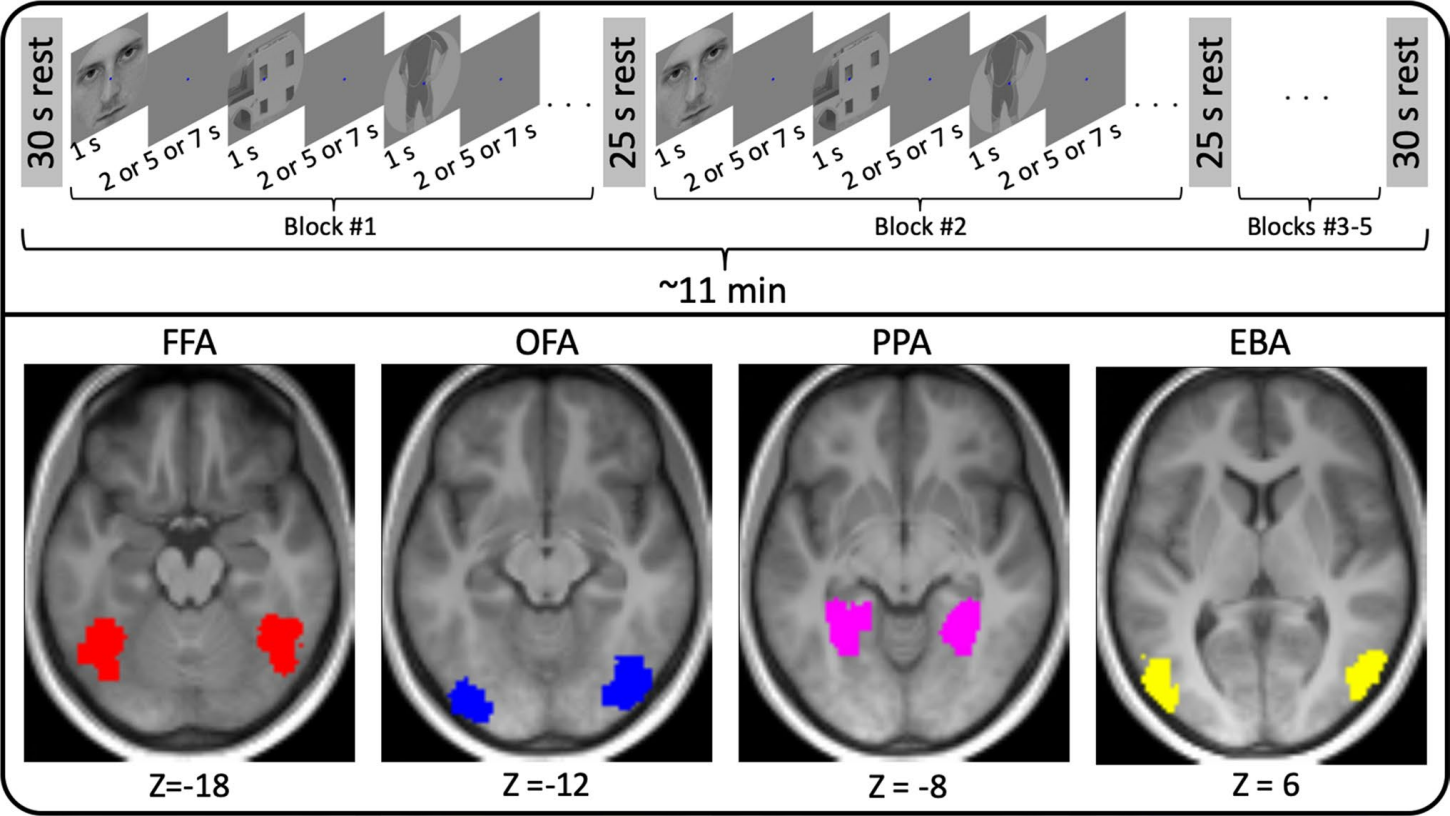
Illustration of the visual stimulus paradigm (top) and depiction of ROI locations (bottom)

The above fMRI data were used to capture brain activity in response to a blocked event-related visual task paradigm (Fig. 1) [20]. Volunteers viewed grayscale images of faces, houses or headless bodies that were displayed centrally, subtending 3.8 × 3.8° on a uniform gray background. The visual stimuli were presented via an MRI-compatible LCD screen (32′ NNL LCD Monitor, NordicNeuroLab, Bergen, Norway; refresh rate 60 Hz) which was placed at 142 cm from the observer. For each ~ 11-min fMRI data acquisition with one of the MB factors, stimuli were presented in five ~ 1.5-min blocks with 30 s rest before the first block and after the last block and 25 s rest periods between the blocks. The visual stimuli were separated by 2, 5 or 7 s and then presented for 1 s. Both the presentation order of the stimuli and the interstimulus interval were pseudo randomized in each of the blocks but the stimuli were identical for all participants and all three MB factors. The participants were instructed to fixate on a dot in the center of the visual display during the entire experiment. During the stimulus presentation, no response was requested from the volunteers. To ensure their attentiveness, after the fMRI data acquisition, they were presented with a group of photos and quizzed whether the photos were new or seen during the fMRI paradigm. Stimulus presentation was controlled by MATLAB R2015a (The MathWorks Inc., Natick, MA, USA) using PTB-3 (http://psychtoolbox.org/credits).

### Data analysis

All analyses were performed in Matlab (MathWorks, Natick, MA, USA) relying on the SPM12 software package [19] and custom-made Matlab scripts. Each of the three fMRI data sets with either MB1, MB4 and MB6 was fed through an identical preprocessing pipeline and subsequent statistical analysis steps with a general linear model (GLM) at the individual-level and each of the five different variants of the haemodynamic response function (HRF) in SPM. The final group-level effect was calculated via a random-effect design either voxel-wise or as an average within each of the four ROIs that were identified with the help of independent functional localizer scans (further details below).

#### Pre-processing

Before statistical analysis, each fMRI dataset was realigned to the first volume of each time-series, co-registered to the anatomical image of the same individual (via the mean of the realigned and unwarped time-series) and normalized to MNI152 space before being smoothed with a 6 mm FWHM 3D Gaussian kernel. As part of the GLM, high-pass filtering and pre-whitening were also implemented. Each data set was also segmented into tissue components and the GM segment was thresholded to include those above a probability of 0.1 [24].

#### Haemodynamic response functions and effective β’s

Five commonly used, [19, 25, 26] built-in HRF models in SPM12 were independently fitted to the each of the three time-series (MB1/4/6). Namely, we employed

- the canonical HRF model (CAN)
- the canonical HRF model plus its temporal derivative (TD)
- the canonical HRF model plus both its temporal and dispersion derivatives (DD)
- the finite impulse response model (FIR)
- a combination of three gamma functions (GAM)

Importantly, these models provide varying flexibility by relying on different sets of basis functions that vary in number and shape. Furthermore, CAN is widely adopted in fMRI studies owing to its simple interpretability. Including temporal and dispersion derivatives enable the consideration of small variation in the timing and duration of the hemodynamic response, respectively. Finally, FIR and GAM are more flexible models for GLM fit with the former fitting to each time point from the onset of the stimulus a separate delta function.

Because these models differ in complexity and hence in the number of parameter estimates each of them yields through the GLM fit, direct comparison and interpretation are nontrivial. Therefore, for each of the models, we computed a single descriptive statistic, *H*, as an estimate of the HRF amplitude and used it in subsequent statistical analyses.

The CAN model yields a single parameter which is simply taken as *H*. Adding the derivative term to the CAN model will require one additional term but make the model more robust against slight time variation in BOLD response. Fitting both terms but using only the non-derivative term may induce amplitude bias due to the delay difference between the model and the measured time series. To counteract this effect, the estimated amplitude is calculated by incorporating the derivative term [27]. Therefore, for the TD model, we set

1 $$H = \mathrm{sign}\left({\widehat{\beta }}_{1}\right)\sqrt{{{\widehat{\beta }}_{1}}^{2}+{{\widehat{\beta }}_{2}}^{2}},$$

where ${\widehat{\beta }}_{1}$ is the estimated parameter for the canonical HRF and ${\widehat{\beta }}_{2}$ is for the temporal derivative term. Similarly, for DD, we set [28]

2 $$H = \mathrm{sign}\left({\widehat{\beta }}_{1}\right)\sqrt{{{\widehat{\beta }}_{1}}^{2}+{{\widehat{\beta }}_{2}}^{2}+{{\widehat{\beta }}_{3}}^{2}},$$

where ${\widehat{\beta }}_{1}$ is the estimated parameter for the canonical HRF, while ${\widehat{\beta }}_{2}$ and ${\widehat{\beta }}_{3}$ are for the temporal and dispersion derivative terms, respectively. For the GAM model, we used the same approach and set

3 $$H = \mathrm{sign}\left({\widehat{\beta }}_{1}\right)\sqrt{{{\widehat{\beta }}_{1}}^{2}+{{\widehat{\beta }}_{2}}^{2}+{{\widehat{\beta }}_{3}}^{2}},$$

where ${\widehat{\beta }}_{1}$, ${\widehat{\beta }}_{2}$ and ${\widehat{\beta }}_{3}$ are the estimated parameters for the three fitted gamma functions. For FIR, we calculated the average of the estimated parameters assigned to the interval ranging between 4 and 6 s from the stimulus onset as

4 $$H=\frac{1}{n}\sum_{i=t}^{t+n}{\widehat{\beta }}_{i},$$

where *n* is the number of averaged estimated parameters ($n=\mathrm{roundup}\left(\frac{2}{TR}\right)+1),$ *t* is the index of the first basis function which is at least 4 s from the stimulus onset and the subscript *i* in ${\widehat{\beta }}_{i}$ counts the estimated parameters of the FIR model consecutively such that the (*t* + *n*)th parameter is no more than 6.15 s from the stimulus onset.

The investigated interval for averaging was defined based on the individual HRF estimations using FIR, considering each subject and MB factor to include a period around the peak (Supplementary Fig. 1).

#### Regions of interest

Four category-selective visual cortical ROIs (Table 2), namely the fusiform face area (FFA), [29] occipital face area (OFA), [30] parahippocampal place area (PPA) [31] and extrastriate body area (EBA), [32] were defined individually based on anatomical landmarks and first-level statistical contrast maps (thresholded at *p* < 0.001, uncorrected) of the independent functional localizer scans. The GLM for these statistical contrast maps relied on the canonical HRF. The ROIs were identified as responding to faces vs houses (FFA and OFA), houses vs faces (PPA) or bodies vs houses (EBA). The PPA and EBA were identified bilaterally in all participants, the FFA bilaterally in 16 participants, and OFA in 14 participants. For the ROI-based analyses, a sphere with a radius of 6 mm and a center located at the peak voxel of the identified ROI was defined for each subject.

**Table 2:** Peak voxel MNI coordinates for the four ROIs

ROI	Hemisphere	x (mm)	y (mm)	z (mm)	N
FFA	Right	43 ± 1.1	− 55 ± 1.2	− 18 ± 1.0	16
	Left	− 40 ± 0.9	− 57 ± 1.5	− 17 ± 0.9	16
OFA	Right	39 ± 1.1	− 77 ± 1.3	− 12 ± 1.0	14
	Left	− 38 ± 1.2	− 82 ± 2.2	− 11 ± 1.0	14
EBA	Right	51 ± 0.7	− 71 ± 0.9	2 ± 1.2	17
	Left	− 48 ± 1.0	− 75 ± 1.6	6 ± 1.6	17
PPA	Right	29 ± 0.6	− 48 ± 1.0	− 7 ± 0.4	17
	Left	− 28 ± 0.9	− 48 ± 1.1	− 8 ± 0.4	17

MNI coordinates (*x*, *y*, *z* in millimeters) are provided separately for both hemispheres and each investigated ROI. Provided data are mean ± standard error across the number of participants (*N*) for whom these regions were individually identifiable using *p* < 0.001 uncorrected threshold

*FFA* fusiform face area, *OFA*  occipital face area, *PPA* parahippocampal place area, *EBA* extrastriate body area

#### Group-level effect

Voxel-wise group-level random-effects analyses were performed on individual effect sizes of the variable H (i.e. HRF amplitudes resulted in the GLM fit in Eq. 1–4) for the main effect (i.e. face + house + body vs baseline) as well as separately for face, house and body for each multi-band factor and HRF model. The resulting *t* value maps were thresholded at a false positive rate, *α*, of *p* < 0.001 (uncorrected) and multiplied by the GM mask.

Separate group-level effects were also obtained by calculating the average effect size, H, within each of the ROIs (averaged across the hemispheres) for the contrast that is relevant for the ROI (face versus baseline for FFA and OFA, house versus baseline for PPA and body versus baseline for EBA). The results within the ROIs are based on averaging across the participants to compare HRFs as well as averaging across both subjects and HRFs to compare MB factors.

#### ROI-based and voxel-wise ANOVA

The main effect of MB acceleration and HRF model as well as their interaction were formally investigated with two separate analyses. In one of these analyses, the ROI average of the effect size, *H*, was extracted for each participant for all combinations of the three MB acceleration levels and the five HRF models and inserted into a two-way repeated-measures ANOVA in Matlab via the built in fitrm and ranova functions. Because 12 tests were made, the level of statistical significance was set at 0.05/12 = 0.0042. The 12 tests were the main effects for MB acceleration, main effect for HRF model and their interaction in each of the 4 ROIs. The corresponding voxel-wise analysis was performed in SPM with MB acceleration and HRF model as within-subject factors. Finally, spm_make_contrast([3 5]) provided the contrasts for the voxel-wise analyses of the main effects and the interaction term. Maps were thresholded at *p* < 0.001, uncorrected.

#### Additional outcome measures

Apart from the group-level t statistics, we extracted the adjusted goodness-of-fit (GoF) coefficient (*R*^2^) for GLM fit with the help of the SPM MACS toolbox, [33] as well as the number of grey matter voxels exceeding the threshold for all group statistics. The former was used as a marker for the quality of the GLM fit for each MB factor and applied HRF model. These adjusted *R*^2^ values were evaluated both voxel-wise and within the ROIs by averaging across the participants to compare HRFs or averaging across both subjects and HRFs to compare MB factors. The adjusted *R*^2^ is an improved GoF measure that considers model complexity and penalizes the fit in relation to the number of variables in the model [34].

## Results

### Group-level ROI results

**Fig. 2:**
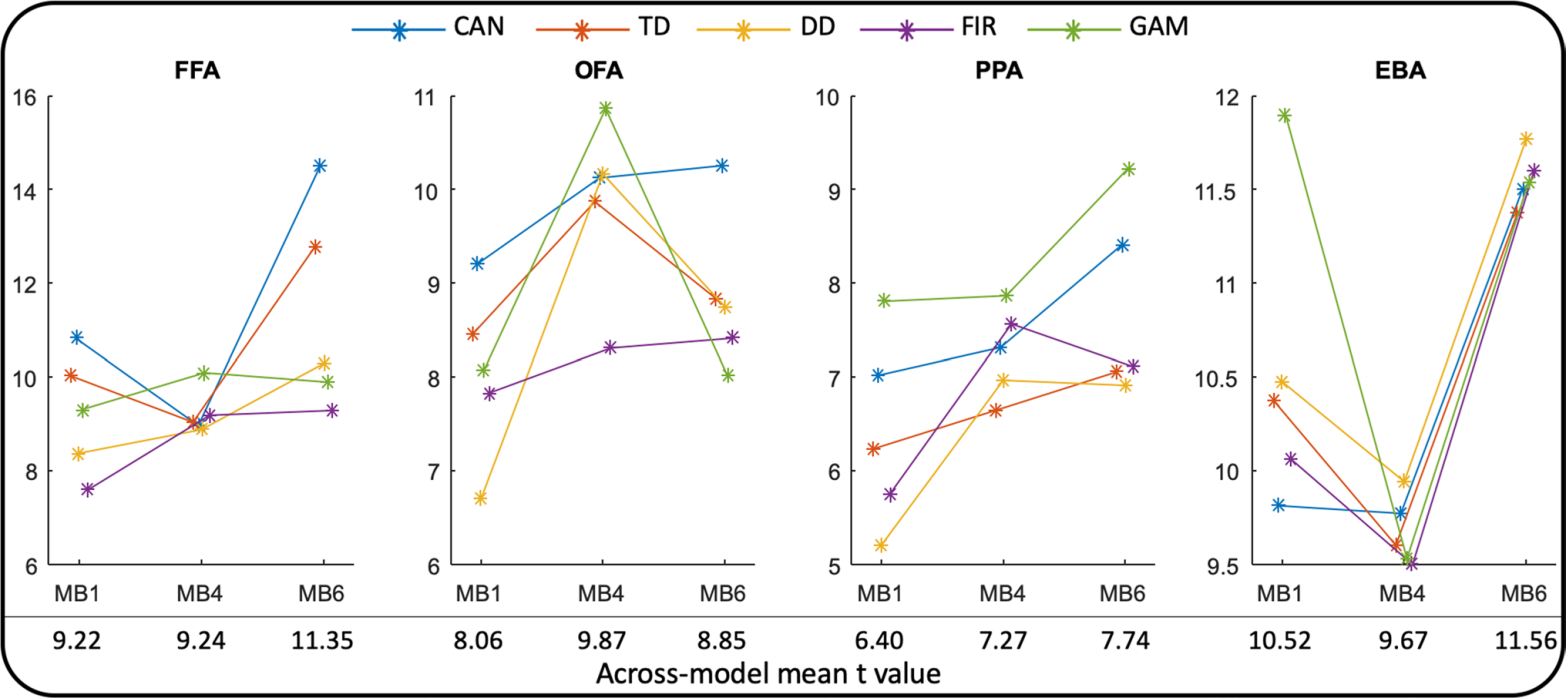
ROI-averaged *t* values (*p* < 0.001 uncorrected) from the group-level random-effects analyses. Four ROIs are shown from left to right for each of the three MB factors. For each data set in each ROI, five different HRFs were used for the analyses. For each of the MB factors, the corresponding *t* values are averaged across HRF models and given below the plots. *CAN* Canonical HRF (blue), *TD* Canonical HRF + its time derivative (red), *DD* Canonical HRF + both its temporal and dispersion derivatives (yellow), *FIR* finite impulse response HRF (purple), *GAM* combination of three gamma functions (green), *FFA* fusiform face area, *OFA* occipital face area, *PPA* parahippocampal place area, *EBA* extrastriate body area

The *t* value for each ROI of the group-level statistical analysis was variable with respect to both anatomical location and HRF for each of the three MB factors but in all cases provided a robust activation with a mean *t* value of at least 5 (Fig. 2). To assess whether the increased temporal sampling rate provides an overall benefit, the group-level *t* values were further averaged across HRFs for an easier comparison of MB factors (listed below each subplot). Although in each ROI either MB4 or MB6 outperformed MB1 on average, the differences were sometimes minimal (e.g. MB1 vs MB4 in FFA), MB1 could be better (e.g. MB1 vs MB4 in EBA) and MB6 was not the overall winner.

### Voxel-wise *t* value maps

**Fig. 3:**
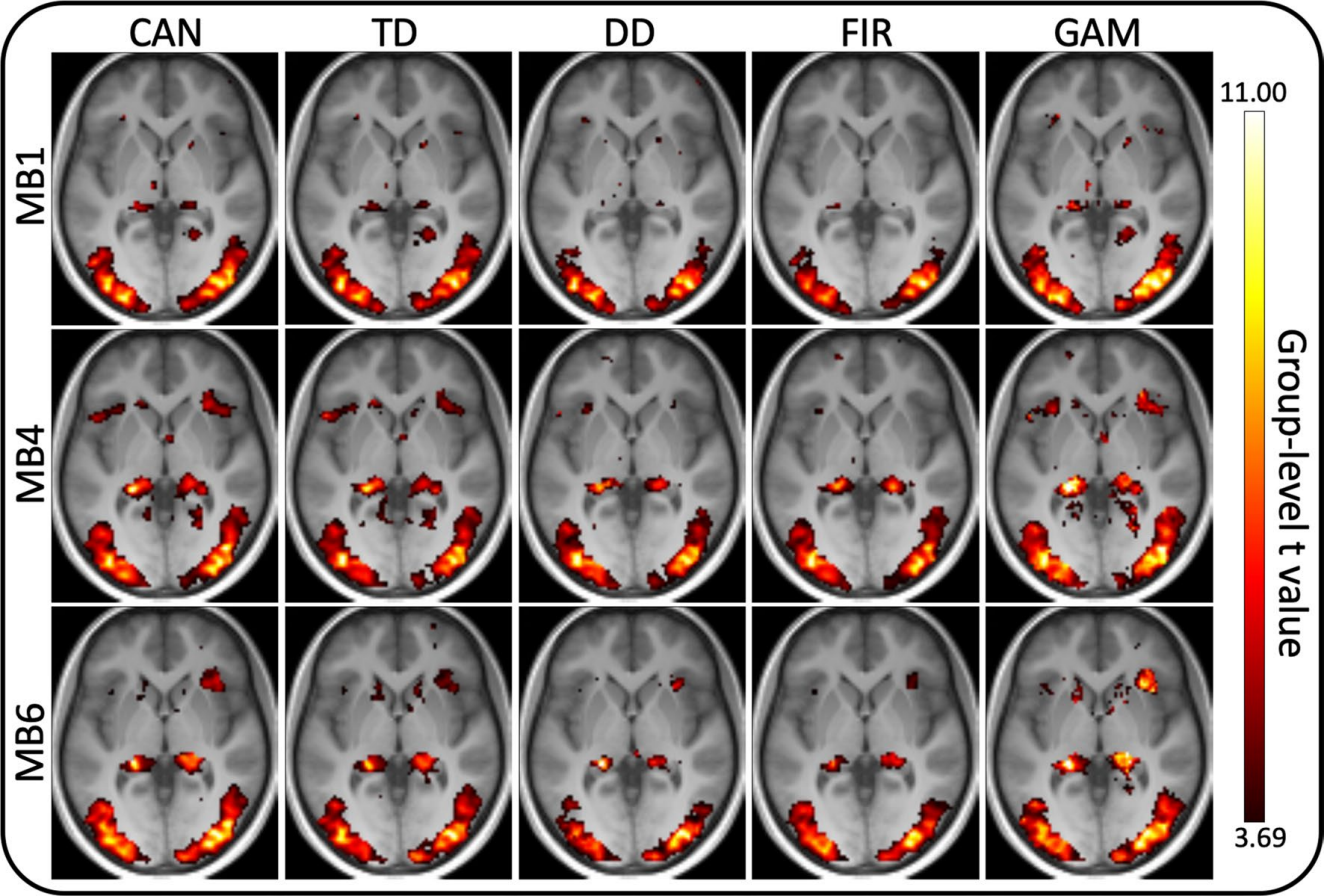
Voxel-wise *t* value (*p* < 0.001 uncorrected) maps from the group-level random-effects analyses, for each of three multi-band factors (rows) and five HRFs (columns). The background image is the axial slice at *z* = 0 in MNI coordinate space. *CAN* Canonical HRF, *TD* Canonical HRF + its time derivative, *DD* Canonical HRF + both its temporal and dispersion derivatives, *FIR* finite impulse response HRF, *GAM* combination of three gamma functions

Supporting the above results from the specific ROIs, voxel-wise comparisons yielded similarly variable group-level *t* values with respect to HRFs and MB factors (Fig. 3). When benefits of MB imaging can be identified, the MB acceleration factor need not be the highest achievable—in this case going from MB1 to MB4 often already provides all the benefit.

### ROI-based and voxel-wise ANOVA

**Fig. 4:**
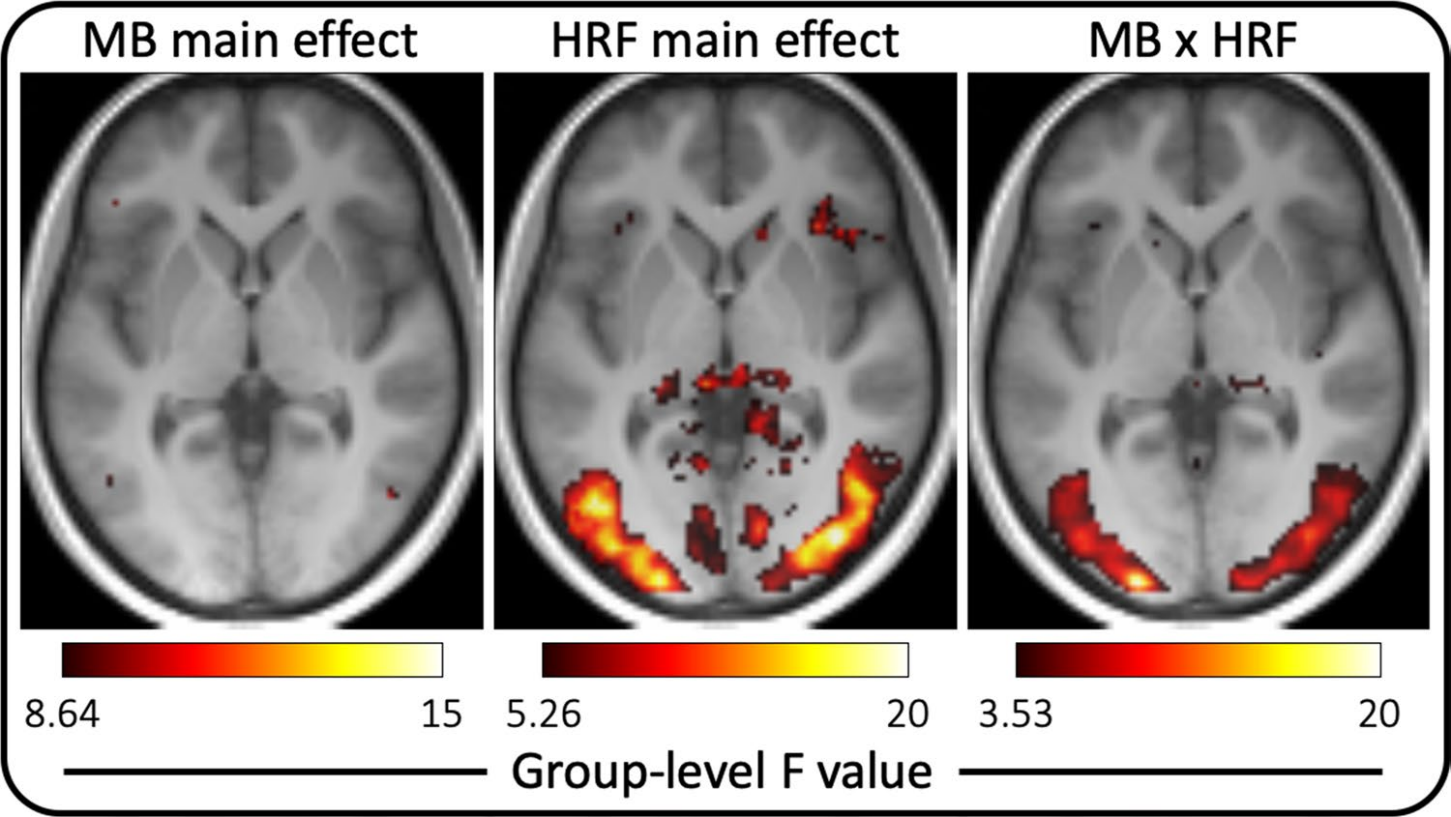
Voxel-wise thresholded *F* value (*p* < 0.001 uncorrected) maps from the group-level two-way repeated-measures ANOVA with the main effect of MB acceleration factor (left, *F*(2,32) = 8.64), the main effect of the HRF model type (middle, *F*(4,64) = 5.26) and their interaction (right, *F*(8,128) = 3.53). The background image is the axial slice at *z* = 0 in MNI coordinate space. Note that the color scaling is different for the three subplots and were chosen in attempt to display the results without clipping or missing voxels

In summary, these analyses formalized and corroborated the above findings (Figs. 2, 3) in that main effect for HRF model is a consistent finding while the main effect of MB acceleration is only a weak predictor of group-level outcome. More specifically, in ROI analysis the main effect of the HRF model and the interaction of MB factor and HRF model were statistically significant at *p* < 0.0001 for each of the four ROIs (Table 3). The main effect of MB factor was somewhat variable with *p* = 0.0915/0.2137/0.1468/0.0049 in FFA, OFA, EBA and PPA, respectively. Note that without Bonferroni correction the main effect of MB acceleration would be statistically significant in the PPA. Nevertheless, the results of the corresponding voxel-wise SPM analysis (Fig. 4) indicated only a main effect for the HRF model type but not the MB acceleration factor, which suggests that the significant ROI-level finding without Bonferroni correction would likely be a false positive. A smaller effect for the interaction of these two variables could also be detected.

**Table 3:** Results of the ROI-level ANOVA

	FFA	OFA	PPA	EBA
MB	*F*(2,30) = 2.59 *p* = 0.0915	*F*(2,26) = 1.64 *p* = 0.2137	*F*(2,32) = 6.31 *p* = 0.0049	*F*(2,32) = 2.04 *p* = 0.1468
HRF	*F*(4,60) = 24.97 *p* < 0.0001	*F*(4,52) = 14.78 *p* < 0.0001	*F*(6,64) = 21.46 *p* < 0.0001	*F*(4,64) = 41.15 *p* < 0.0001
MBxHRF	*F*(8,120) = 15.82 *p* < 0.0001	*F*(8,104) = 13.11 *p* < 0.0001	*F*(8,128) = 16.97 *p* < 0.0001	*F*(8,128) = 34.75 *p* < 0.0001

Both the *F* value, degrees of freedom and the *p* value are provided for the main effects of MB acceleration (top row) and HRF model type (middle row) as well as their interaction (bottom row) in each of the 4 ROIs (columns)

*FFA* fusiform face area, *OFA* occipital face area, *PPA* parahippocampal place area, *EBA* extrastriate body area

### Goodness of fit

**Fig. 5:**
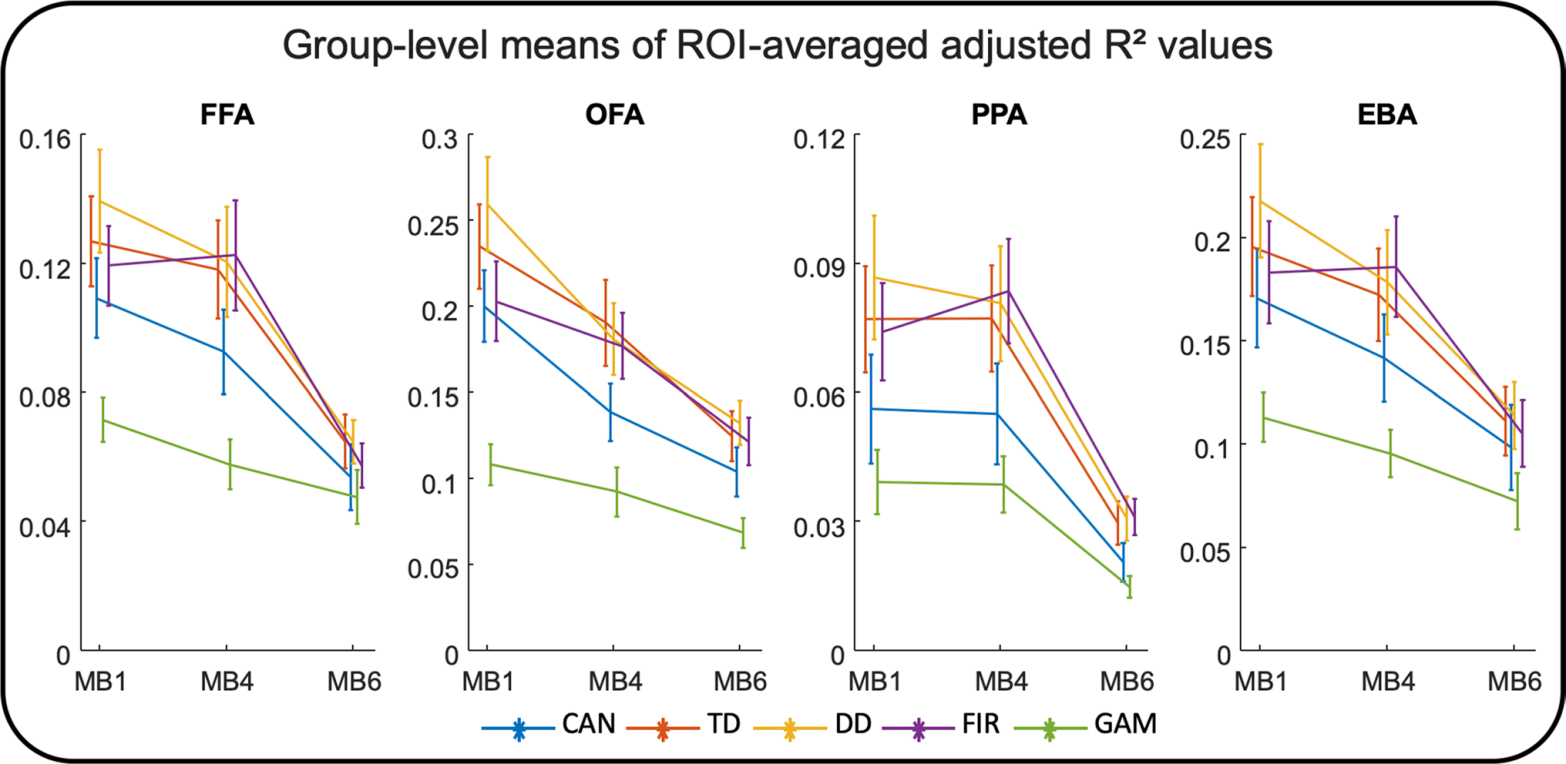
Group-level mean of ROI-averaged adjusted *R*^2^ values for each of three MB factors and five HRFs. As the MB factor is increased from 1 to 4 to 6, the goodness-of-fit (i.e. adjusted *R*^2^) values tend to drop in all observed ROIs. *CAN* Canonical HRF (blue), *TD* Canonical HRF + its time derivative (red), *DD* Canonical HRF + both its temporal and dispersion derivatives (yellow), *FIR* finite impulse response HRF (purple), *GAM* combination of three gamma functions (green), *FFA* fusiform face area, *OFA* occipital face area, *PPA* parahippocampal place area, *EBA* extrastriate body area

Figure 5 provides the adjusted goodness-of-fit values [33] averaged in each ROI and for each HRF for each of the three MB factors. Unsurprisingly, an almost universally monotonic decline is apparent as the MB factor is increased. Given the highly complex physiological mechanism of the BOLD response in comparison to the HRF models that contain only a few parameters, as more data are collected the more apparent the difference between the model and the data becomes. Voxel-wise results corroborate these conclusions both when averaged across participants or when averaged both across participants and across HRF models (Supplementary Fig. S2).

### Number of activated voxels

**Fig. 6:**
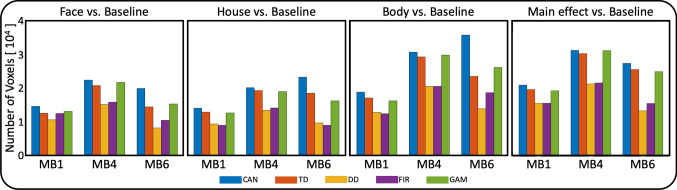
Number of activated voxels (*p* < 0.001 uncorrected) after separate group-level random-effects analyses for face, house and body as well as their combined main effect while utilizing one of five different HRFs. The MB4 acceleration consistently results in the largest number of activated voxels. Notable however is that the choice of HRF can have a larger effect on the number of activated voxels (e.g. CAN on average outperforms DD by a larger margin). *CAN* Canonical HRF (blue), *TD* Canonical HRF + its time derivative (red), *DD* Canonical HRF + both its temporal and dispersion derivatives (yellow), *FIR* finite impulse response HRF (purple), *GAM* combination of three gamma functions (green)

Overall, MB imaging did not produce measurable difference in the number of activated voxels within the GM mask in the four contrasts that were tested (Fig. 6). Indeed, choice of the HRF imparted a larger effect on the number of activated voxels than did the MB acceleration factor.

## Discussion

Through a systematic empirical investigation, we found the impact of increased temporal sampling to be variable when evaluated across several anatomical locations and HRF variants in a group-level random effects analysis of task-based fMRI time-series data. While a benefit could be reasonably expected from increased temporal sampling of a given process, the practical limitations can balance out any such benefit. In the case of the nuclear magnetic resonance phenomenon, the sampling and signal generation processes are intricately intertwined through T1 relaxation, such that increased temporal sampling rate (i.e. shorter TR) results in lower SNR. Therefore, methods that reduce TR will often coincidentally reduce SNR as well.

Lowering TR is achievable through various means. Although we utilized MB acceleration to maintain full coverage of the brain, it is imperative to state that the fluctuating benefit of the increased temporal sampling rate (e.g. see Fig. 2) is unlikely to be unique to MB imaging per se. For example, TR can be shortened by acquiring fewer slices in a multi-slice 2D EPI acquisition—imparting a similar SNR hit.

Previous output highlighted the fact that it is not always the higher MB acceleration factor that provides the best statistical outcome [35] and indicated a similar reduction of the mean *t* value in subject specific ROIs and/or the number of activated voxels in a task-based fMRI [10, 11]. As these studies used different stimuli and/or different ROI(s), our results corroborate and widen the scope of their finding. Additionally, the explicit variation of HRF in the present work further supports the conclusion that the impact of temporal sampling can be dependent on several other acquisition or processing parameters.

We hypothesized that the choice of the HRF would impact the outcome of the group-level studies and modulate the effect of the MB factor, because the temporal evolution of the hemodynamic response varies with anatomical location in a given person [17, 36] as well as among individuals [18]. Therefore, fitting either HRF to the local time-series data will be imperfect to different degrees. Additional data, with higher temporal sampling rate, cannot be expected to impact each model identically.

The separate systematic evaluation of the adjusted GoF measure for each fit supports this notion (Fig. 4). Although, it is often taken for granted that additional data will improve results of regression, this should only be expected when the assumed model correctly captures all details of the process. Otherwise, the additional data will not improve the reliability of the fit and rather serve to pinpoint that the assumed model is not appropriate. Indeed, with additional data from MB4 the fit improves for some models in some anatomical areas (e.g. FIR in FFA) but not all, while the fit for data with MB6 universally worsened. Note that the adjusted GoF measure penalizes higher number of variables in a model, therefore even FIR (the most flexible model) does not improve with the additional data.

MB imaging can also be used to reduce total acquisition time. Although, this practice does not increase statistical degrees of freedom but the increased patient comfort may results in fewer and/or smaller motion artifacts. This aspect was not investigated here.

## Conclusions

Increasing the temporal sampling rate of task-based fMRI data does not provide a universal benefit over the entire brain and outcome depends also on the choice of HRF in the statistical analysis pipeline. Given that all MB factors and HRFs provided robust activations, these results caution against the default use of acquisition methods with the highest possible sampling rate (i.e. lowest TR). Rather, a practically implementable recommendation is to perform a small pilot study with various TRs for the specific stimulus and cohort to identify the most appropriate settings for the full experiment involving the entire group of participants.

## Supplementary Information

Below is the link to the electronic supplementary material.

**Supplementary Figure S1.** Confirmation of the appropriate usage of the FIR HRF in the group-level analysis. Time course of the fitted FIR HRF for both hemisphere (columns) and each MB factor (rows) in each ROI. The time courses were extracted from the centre of each ROI and point-wise averaged across the entire group. In this study, the mean amplitude of the FIR between 4–6 s from each participant was taken to the group-level analysis. *FFA* fusiform face area, *OFA* occipital face area, *PPA* parahippocampal place area, *EBA* extrastriate body area (TIFF 11971 KB)

**Supplementary Figure S2.** Group-level voxel-wise adjusted *R*^2^ values of the GoF evaluation are given after averaging across participants (top) or after averaging across both participants and the separate evaluations with the five different HRF models. These voxel-wise results corroborate the evaluations within the ROIs as given in Fig. 4. All axial slices are displayed at *z* = 0, while the sagittal and coronal slices on the bottom are at *x* = 42 and *y* =  − 74, respectively. *CAN* Canonical HRF, *TD* Canonical HRF + its time derivative, *DD* Canonical HRF + both its temporal and dispersion derivatives, *FIR* finite impulse response HRF, *GAM* combination of three gamma functions (TIFF 8853 KB)
