# Supplementary figures and images for: An empirical investigation of the benefit of increasing the temporal resolution of task-evoked fMRI data with multi-band imaging
Source: MAGMA. 2021 Mar 25;34(5):667–76. doi: 10.1007/s10334-021-00918-z (PMC8421273; doi:10.1007/s10334-021-00918-z)

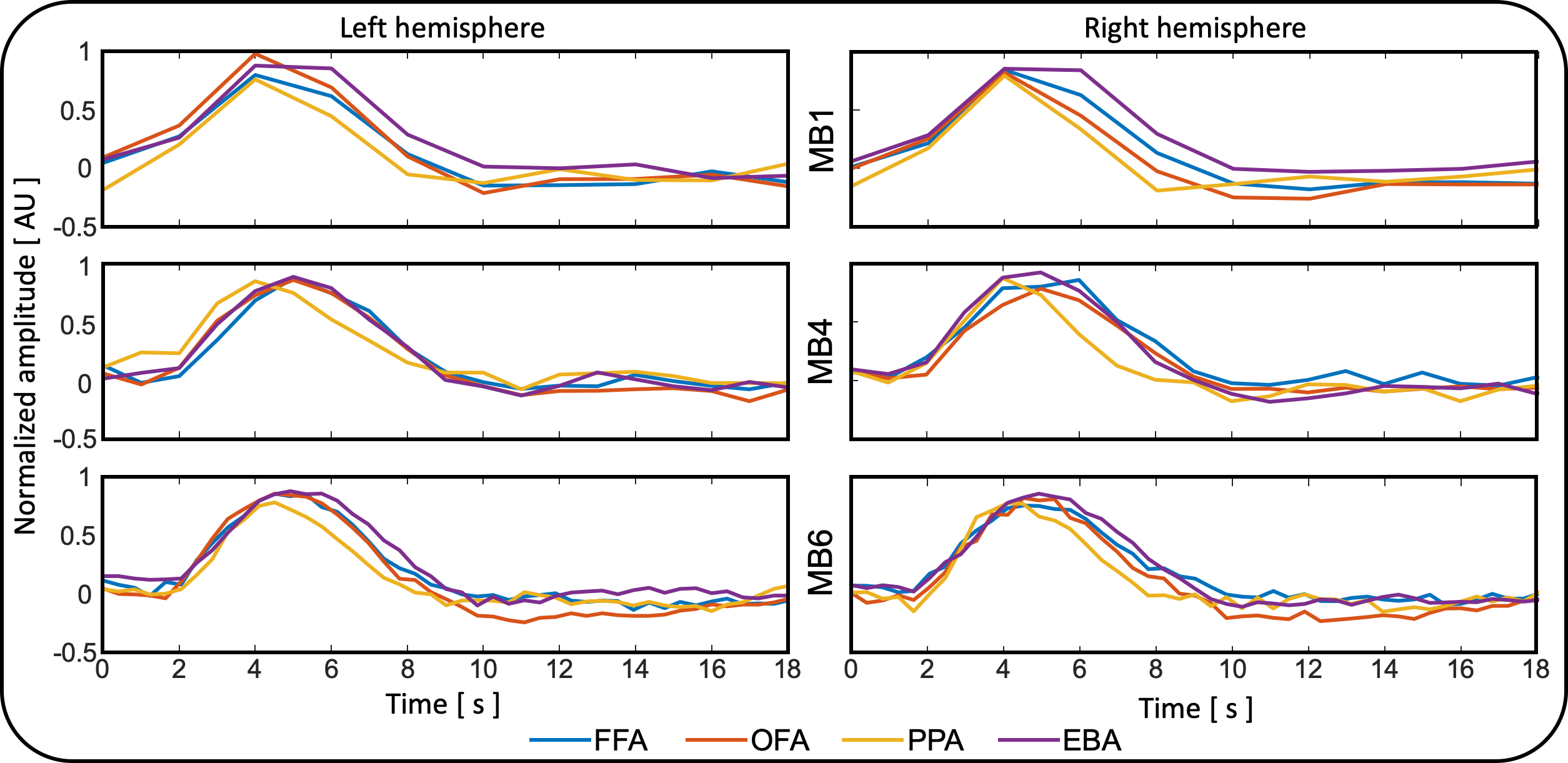

Supplement: Supplementary file 1 — Supplementary Figure S1. Confirmation of the appropriate usage of the FIR HRF in the group-level analysis. Time course of the fitted FIR HRF for both hemisphere (columns) and each MB factor (rows) in each ROI. The time courses were extracted from the centre of each ROI and point-wise averaged across the entire group. In this study, the mean amplitude of the FIR between 4–6 s from each participant was taken to the group-level analysis. FFA fusiform face area, OFA occipital face area, PPA parahippocampal place area, EBA extrastriate body area (TIFF 11971 KB) [file 10334_2021_918_MOESM1_ESM.tiff]

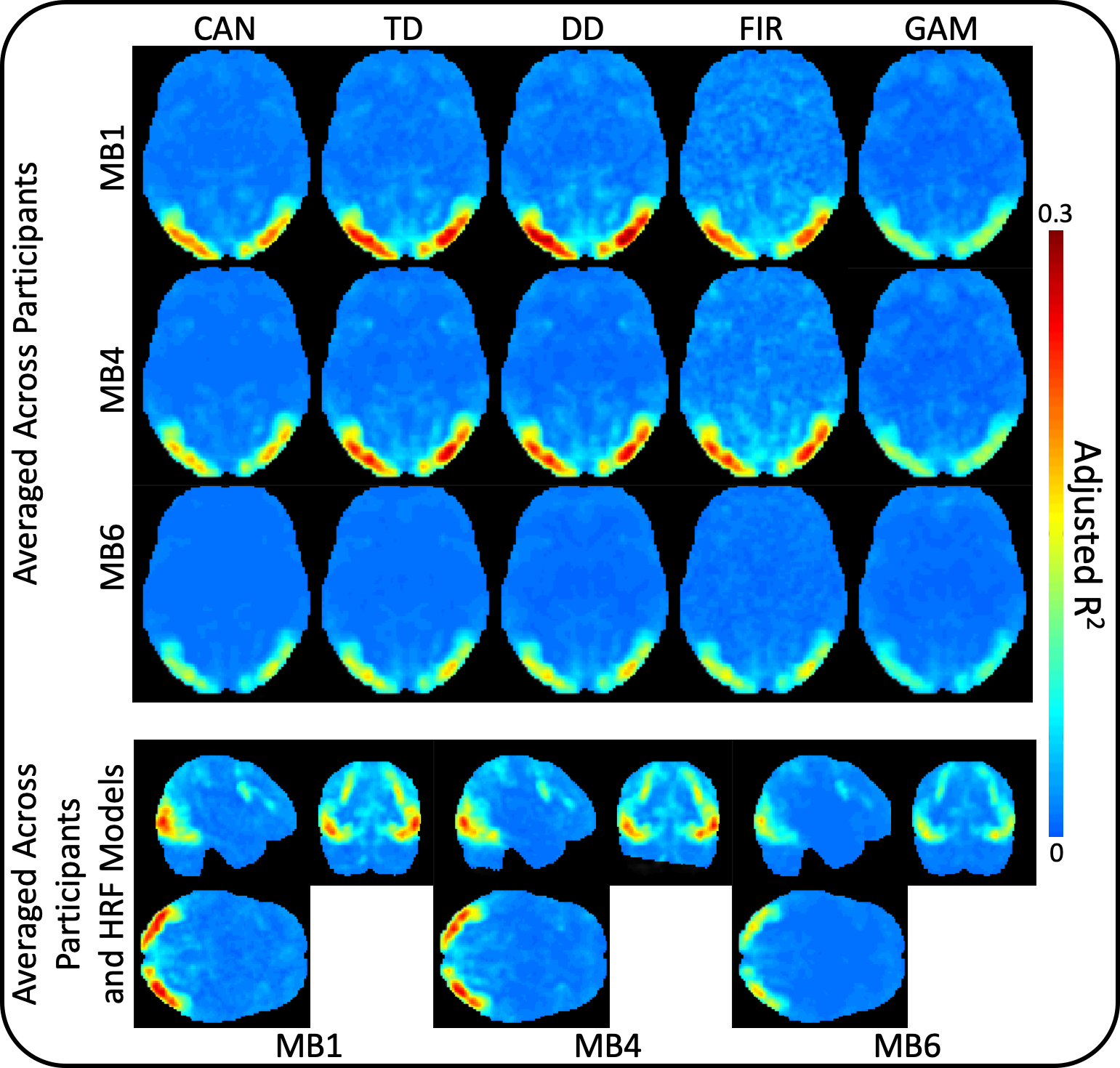

Supplement: Supplementary file 2 — Supplementary Figure S2. Group-level voxel-wise adjusted R2 values of the GoF evaluation are given after averaging across participants (top) or after averaging across both participants and the separate evaluations with the five different HRF models. These voxel-wise results corroborate the evaluations within the ROIs as given in Fig. 4. All axial slices are displayed at z = 0, while the sagittal and coronal slices on the bottom are at x = 42 and y = − 74, respectively. CAN Canonical HRF, TD Canonical HRF + its time derivative, DD Canonical HRF + both its temporal and dispersion derivatives, FIR finite impulse response HRF, GAM combination of three gamma functions (TIFF 8853 KB) [file 10334_2021_918_MOESM2_ESM.tiff]
